# The impact of Covid-19 on student achievement: Evidence from a recent meta-analysis^☆^

**DOI:** 10.1016/j.edurev.2023.100530

**Published:** 2023-05

**Authors:** Giorgio Di Pietro

**Affiliations:** a European Commission- Joint Research Centre^1^, Edificio Expo, Calle Inca Garcilaso, 3, 41092, Seville, Spain; b Institute of Labour Economics (IZA), Schaumburg-Lippe-Straße 5-9, 53113, Bonn, Germany

**Keywords:** Covid-19, Student achievement, Meta-analysis, Heterogeneity, Learning deficit

## Abstract

This work attempts to synthetize existing research about the impact of Covid-19 school closure on student achievement. It extends previous systematic reviews and meta-analyses by (a) using a more balanced sample in terms of country composition, (b) considering new moderators (type of data and research design), and (c) including studies on tertiary education students in addition to primary and secondary education students. Our meta-analysis findings show that the pandemic had, on average, a detrimental effect on learning. The magnitude of this learning deficit (about 0.19 standard deviations of student achievement) appears to be roughly comparable to that suffered by students who have experienced a significant disruption in their schooling due to a major natural disaster (e.g., Hurricane Katrina). Students are also found to have lost more ground in math/science than in other subjects. Additionally, one year or more after the first lockdown, students seem to have been unable to catch up on unfinished learning from the pandemic. This result suggests that more efforts should be made to ensure students recover their missed learning in order to avoid negative long-term consequences for them and society.

## Highlights

• We perform a meta-analysis to study the effect of Covid-19 on student achievement.
• Our dataset includes 239 estimates from 39 studies covering 19 countries.
• The pandemic had an overall negative effect on learning outcomes.
• Students lost more ground in math/science than in other subjects.
• One year or more after Covid-19 students have not recovered from the initial learning loss.

## 1 Introduction

The Covid-19 pandemic caused a major disruption in the schooling system around the world. In most countries, educational institutions had to close for several weeks or months in an attempt to reduce the spread of the virus (UNESCO, 2020a). Students had to continue their schooling from home using different learning tools such as video conferencing, radio and TV. However, the outbreak of Covid-19 was so sudden that there was little or no time for many schools to design and implement learning programs specifically designed to support children's learning while at home. A significant proportion of teachers were unprepared for online learning as they lacked appropriate pedagogical and digital skills (School Education Gateway, 2020). Similarly, many students also struggled to adjust to the new format of learning. In addition to problems in accessing appropriate technology (computers, reliable internet connection, etc.), not all students had a home environment free of disturbances and distractions, hence conducive to learning (Pokhrel & Chhetri, 2021). A large number of parents had serious difficulties in combining their work responsibilities (if not joblessness) with looking after and educating their children (Soland et al., 2020). Moreover, there is evidence showing that Covid-19 and the related containment measures have had a detrimental effect on children's wellbeing (Xie et al., 2020). Longer periods of social isolation might have adversely affected students' mental health (e.g., anxiety and depression) and physical activity (Vaillancourt et al., 2021). This is also likely to have contributed to negatively impact their academic performance given the close association between mental and physical health and educational outcomes (Joe et al., 2009).

While in the literature there is already a relatively large consensus that student learning suffered a setback due to Covid-19, as pointed out by several researchers (e.g., Donnelly & Patrinos, 2022; Patrinos et al., 2022), more research in this area is still needed. Findings from new studies are important given that, as stated in a recent article published in the World Economic Forum, the full scale of the impact of the pandemic on the education of children is “only just starting to emerge” (Broom, 2022). Not only is a better understanding of the educational impact of Covid-19 needed, but special attention should be paid to investigate the legacy effects of the pandemic. As argued in several papers (e.g., Hanushek & Woessmann, 2020; Psacharopoulos et al., 2021), there is the risk that the disruption in learning caused by Covid-19 may persist over time, having long-term consequences on students’ knowledge and skills as well as on their labour market prospects. It is therefore very important to determine if and to what extent those children whose schooling was disrupted by Covid-19 subsequently got back on track and reduced their learning deficits.^2^ Similarly, it is relevant to gain a more solid understanding of how the educational impact of Covid-19 varies across students and circumstances. This would help educators and policymakers identify those groups of students who may need extra support to recover from the learning deficit caused by the pandemic.

This paper uses meta-analysis in an attempt to synthetize and harmonize evidence about the effect of Covid-19 school closures on student learning outcomes. Meta-analysis, which is widely employed in education as well as in other fields, combines the findings of multiple studies in order to provide a more precise estimate of the relevant effect size and explain the heterogeneity of the results that have been found in individual studies. A total of 239 separate estimates from 39 studies are considered. We extend previous systematic reviews and meta-analyses^3^ in four main ways. First, compared to previous meta-analyses, this study covers a larger number of countries (i.e., 19). Not only are several new countries considered in the analysis (e.g., Slovenia, Egypt), but US and UK studies do not dominate the collected empirical evidence. For instance, while in Betthäuser et al. (2023) about 71.1% of the effect sizes are derived from these studies, in our paper the corresponding figure is approximately 33.9%.^4^ This makes our results of more general relevance.^5^ Second, the current meta-analysis adds to previous meta-analyses by including also studies looking at the impact of Covid-19 among tertiary education students in addition to primary and secondary education students. This is important because, as individuals progress through the education system, academic challenges increase and so does the pressure to perform well. Several studies from various countries (e.g., Bratti et al., 2004; Dabalen et al., 2001; Koda & Yuki, 2013) show that the final grade awarded to students successfully completing university is an important predictor of their labour market prospects. Third, while some relevant moderator variables have already been noted (e.g., subject, level of education, geographical area), the present meta-analysis adds several new ones including type of data and research design. The relevance of these factors in explaining the heterogeneity of results across studies is well-known in the meta-analysis literature. For instance, Havránek et al. (2020) indicate that researchers conducting meta-regression analysis in economics should consider data types. Similarly, Stanley and Jarrell (1989) suggest that variables capturing differences in methodology need to be included among moderators in meta-regression models. More in general, moderators are situational variables as well as characteristics of studies that might influence the effect estimate (Judd, 2015). Fourth, in contrast to previous similar meta-analyses (e.g., König & Frey, 2022), we look closely at the issue of the specification of the meta-regression model. As observed by Stanley and Doucouliagos (2012), this is a more relevant problem in meta-analysis than in primary econometric studies given the higher risk of exhausting degrees of freedom in the former than in the latter. Following recent literature (e.g., Di Pietro, 2022), we employ different methods to select the moderator variables to be included in the meta-regression model.

The remainder of the paper is set as follows. Section 2 describes the process of selecting studies and collecting data. It also discusses the empirical approach and the possibility of publication bias. Section 3 reports and discusses the empirical results. Section 4 concludes.

## 2 Method

To perform this meta-analysis, we followed the Preferred Reporting Items for Systematic Reviews and Meta-Analysis (PRISMA) (Moher et al., 2009).

### 2.1 Inclusion criteria

With the purpose of this study in mind, a set of inclusion criteria was defined. They guided the selection of the studies included in this meta-analysis. Specifically, the following four inclusion criteria were used:

● the study should quantitatively examine the effect of Covid-19 on student achievement in primary, secondary or tertiary education. This means that the data used in this study were collected before and during the pandemic (or only during the pandemic if, when schools were closed, some students were still receiving in-person teaching thereby simulating pre-pandemic conditions), therefore clearly distinguishing between a control and a treated group, respectively.
● the study should use objective indicators (e.g., test scores) to measure student achievement.
● the study should be based on real data.
● the study should report data on an effect size (or sufficient information to compute it) and its standard error (or *t*-statistic, or *p*-value, or sufficient information to calculate it).

### 2.2 Search ad screening procedures

To identify the relevant studies, we searched in six different electronic databases (i.e., Google Scholar,^6^ EconLit, ScienceDirect, Education Resources Information Center, JSTOR and Emerald). The following keywords were used: “Covid-19 (OR coronavirus OR pandemic OR Cov) AND student (OR academic OR scholastic) performance (OR achievement OR learning OR outcome) OR test score”.

**Fig. 1:**
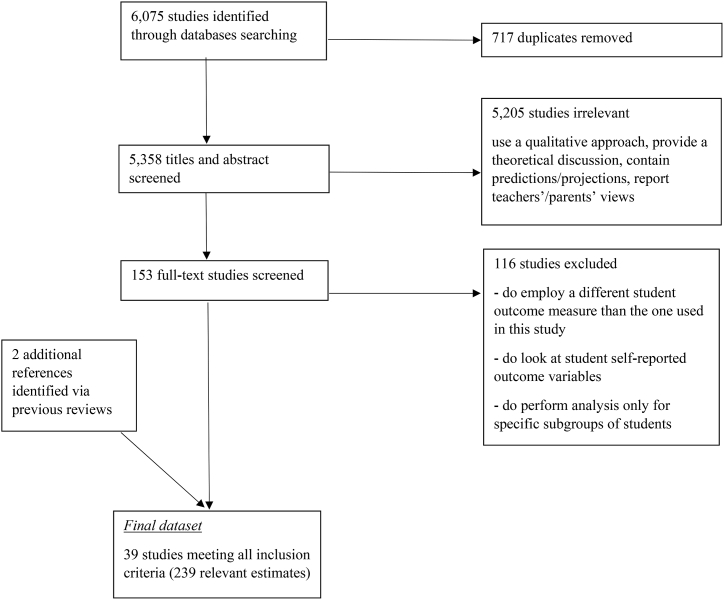
Flow chart of the search and screening process.

This search, which ended on 15^th^ July 2022, delivered 6,075 hits. 717 duplicates were removed. We kept updated or published versions of any working paper we found. Next, the titles and the abstracts of the remaining 5,358 records were assessed. Following this, 5,205 studies were excluded as they use qualitative approaches (e.g., interviews), report teachers'/parents’ views about the educational impact of Covid-19 (e.g., Kim et al., 2022; Lupas et al., 2021), or provide a theoretical discussion about how the pandemic is likely to affect education (e.g., Di Pietro et al., 2020). Similarly, studies containing predictions and/or projections were also removed (e.g., Kuhfeld et al., 2020a). After this initial screening, the content of the remaining 153 studies was carefully examined, and only those fulfilling all the inclusion criteria were considered. In this phase, we excluded studies that, although attempting to understand how the pandemic impacted student learning, employ a different outcome measure (e.g., dropout rate) than the one considered in this meta-analysis (e.g., Tsolou et al., 2021). In the same vein, we removed studies using student self-reported outcome measures as well as those examining the educational impact of Covid-19 on specific subgroups of students (e.g., Agostinelli et al., 2022). Finally, in order to ensure that key sources were not missed, we also screened the references included in previous meta-analyses and systematic reviews. Two more relevant articles were identified through this search. A total of 39 studies was included in this study. Fig. 1 summarizes the literature search and the screening procedure.

While all the titles and abstracts were screened only by the author, the next stages of the study selection process were carried out by the author and by another researcher who independently classified the studies as relevant and irrelevant based on the predefined inclusion criteria. While the inter-rater agreement was very high (i.e., 97%), studies on which there was disagreement were discussed in depth until consensus was reached.

### 2.3 Study coding

All the studies included in this meta-analysis were read in-depth, and relevant information and findings were extracted. Study coding was performed following the same procedure used for the final stages of the study selection process. The inter-rater agreement was again high (i.e., 93%).

In line with the current best practice in meta-analysis (Polák, 2019), we use all relevant estimates included in the selected studies. As argued by Cheung (2019), not doing so results in missed opportunities to take advantage of all the available data to answer the research question/s under investigation. However, a fundamental issue with this approach lies in the dependence between multiple estimates from the same study given that effect sizes are assumed to be independent in meta-analysis (Cheung & Vijayakumar, 2016). As discussed later in the paper, several methods are used to account for within-study dependence.

#### 2.3.1 Effect size calculation

In order to be able to aggregate the various impact estimates reported in the selected studies, one needs to convert them into a common metric. Consistent with previous relevant systematic reviews and meta-analyses, we use the Cohen's *d* as a scale-free effect size measure. Cohen's *d* refers to standardised mean differences and is calculated by dividing the mean difference in student performance between pre-Covid and Covid periods by the pooled standard deviation. While in some cases the Cohen's *d* was retrieved from the studies, in others it was calculated using information directly available from them. Where the latter was not possible, the studies' author/s was/were contacted to obtain the relevant data. If not reported, the Cohen's *d* standard error was computed using the formula given in Cooper and Hedges (1994). In case no information on sample sizes were available from the studies but exact *p*-values were instead reported, the formula provided by Higgins and Green (2011) was employed to obtain standard errors. In some instances, we also used information on effect sizes contained in the electronic supplement of the meta-analysis article by König and Frey (2022). For instance, this was the case when a study does not report Cohen's *d* but this information has been already collected by König and Frey who have contacted the relevant author/s.

#### 2.3.2 Moderator variables

For each effect size, we code several moderator variables, that is, factors potentially influencing the size of the effect of Covid-19 on student achievement. These moderator variables can be divided into two categories: 1) context and 2) characteristics. Regarding the former, we consider:

a) *The level of education.* Several arguments suggest that remote schooling is more challenging for younger students compared to their older counterparts. To start with, younger learners are less likely to have access, and be able to independently use digital devices. They may be unable to sign into an online class without assistance, may need help or supervision to perform an online task, and may more easily get distracted. Parental engagement therefore plays a crucial role in the success of younger pupils in an online learning environment. However, even though critical, the supervision required for online schooling while younger children are at home may turn out to be unsustainable for many parents who are at the same time engaged with remote working (Lucas et al., 2020). There is also evidence showing that younger students are less likely to have a quiet space to work at home than their older peers. For instance, Andrew et al. (2020) found that in the UK during the first Covid-19 lockdown while the proportion of primary school students reporting not to have a designated space to study at home was about 20%, the corresponding figure for secondary school students was approximately 10%. Furthermore, children in early grades may especially miss in person teaching as they depend on situational learning (Storey & Zhang, 2021b). A great emphasis is placed on relationships and interactions with others in order to acquire knowledge. Younger learners are also more likely to need movement and exploration, and these are things that one cannot do while sitting at home and looking at a screen (Hinton, 2020). Finally, some studies (Domínguez-Álvarez et al., 2020; Gómez-Becerra et al., 2020) showed that during Covid-19 younger children present more emotional problems than older children. Tomasik et al. (2021) argued that the former group are more likely to have difficulties in coping with socio-emotional stressors associated with the pandemic. Perhaps also as a result of this, there was greater attention to pastoral care than curriculum coverage among primary school students, as opposed to secondary school students (Julius & Sims, 2020).

In an attempt to investigate how the educational impact of the pandemic varies across student age groups, we distinguish between primary, secondary, and tertiary education students.

b) *Subject.* It is often claimed that the effect of the pandemic on student achievement varies depending on the subject being assessed. Specifically, three main arguments have been advanced to suggest that the pandemic has made students lose more ground in math than in other subjects.

First, while the Covid-19 lockdown has called for increased parental involvement in their children's learning, parents often feel they have difficulties in assisting their children in math. Panaoura (2020) looked at parents' perception of how they have helped their children in math learning during the pandemic in Cyprus. She found that parents' lack of confidence or their low self-efficacy beliefs were enhanced during this period. More teachers' guidance and training would have been needed. Using data on Chinese primary school students during Covid-19, Wang et al. (2022) concluded that parental involvement had a positive impact on children's achievement in Chinese and English, but not in math. While parents are likely to be knowledgeable about the learning content of Chinese and English lessons, this may not be the case for math lessons. In daily life, language practice is more used than math practice. Furthermore, parents may be familiar with math methods different from the ones used by teachers (Shanley, 2016).

Second, teaching math in a fully online context is very challenging. Using data from a survey addressed to math lecturers between May and June 2020, Ní Fhloinn and Fitzmaurice (2021) found that most of the respondents agreed that it is harder to teach math remotely. This is partly due to the idiosyncratic nature of this discipline. It is especially difficult for math instructors to adapt their teaching style to online learning conditions. While many of them used to handwrite the material in real time during their lectures, only a small proportion have the technology to continue doing so online. On the other hand, also students may have problems in communicating math online. Not only do students need to learn and accustom themselves to use technology in order to write mathematical symbols, but this is not always possible in online platforms such as chats (Mullen et al., 2021). Online engagement in math is particularly difficult. Involving students in online discussions around an exact science like math may turn out to be very challenging.

Third, the economic and health problems caused by Covid-19 coupled with the sudden shift to online learning are likely to have increased math anxiety among students. This can be defined as a negative emotional reaction that interferes with the solving of math problems (Blazer, 2011, p. 1102). Math anxiety prevents students from learning math because it leads to low self-esteem, frustration, and anger (Fennema & Sherman, 1976). Mamolo (2022) found that the students’ math motivation and self-efficacy decreased during the pandemic. Similarly, Mendoza et al. (2021) and Arnal-Palacián et al. (2022) provided evidence about higher levels of math anxiety experienced by university and primary school students, respectively, during Covid-19.

In light of the above, subjects have been grouped into three different broad categories: math/science, humanities, and a mix category.

c) *Timing of student assessment during Covid-19*. As stated earlier, an important question is the extent to which the pandemic has long-lasting effects on learning outcomes. Several arguments suggest that the negative effect of Covid-19 on student achievement may decline as we move to a later stage of the pandemic. To start with, a number of provisions are likely to have been taken in order to help students catch up after the first lockdown and following the re-opening of schools (at least temporarily). An UNESCO, UNICEF, World Bank and OECD report (2021) showed that in the third quarter of 2020 many countries around the world were planning to adopt support programs with the aim of reducing the learning deficit suffered by students earlier in the year. These programs include increased in-person class time, remedial programs, and accelerate learning schemes. Additionally, one would expect students and their parents to have become more used to remote learning during successive school closures and periods of online classes. Finally, many teachers and schools have probably learned important lessons from the first lockdown. These lessons might have helped them design and implement more effective remote learning measures in the subsequent phases of the pandemic.

However, despite the aforementioned considerations, it is possible that it will take some time before students are able to recover from the learning deficit caused by Covid-19. Students may experience problems in re-engaging with education activities following the re-opening of schools. There is evidence showing that, after several months of remote schooling, students have become more passive ad feel disengaged from their learning (Toth, 2021). The stress and anxiety stemming from the pandemic are likely to have caused a fall in student motivation and morale. The uncertainty of the learning environment under Covid-19 could have also contributed to reduce students’ educational aspirations (OECD, 2020). Additionally, during the academic year 2021–2022, as a result of successive waves and different variants of Covid-19, schools had to face several problems including significant staff shortages, high rates of absenteeism and sickness, and rolling school closures (Kuhfeld & Lewis, 2022). Evidence from the US shows that the pandemic has aggravated the problem of teacher shortage (Schmitt & deCourcy, 2022). Following school re-opening, teachers faced new requirements (e.g., hybrid teaching, more administrative tasks) that added to their already full workloads prior to Covid-19 (Pressley, 2022). This increased their stress levels, which made them more likely to leave their job. While many teachers have quit their job during the pandemic, this reduction in staff has not been fully offset by new hires.

In an attempt to look at how the educational impact of Covid-19 changes over time, we distinguish whether the student learning outcome was assessed in 2020 or 2021.

d) *The geographical area* where the study takes place. We make a distinction between Europe (i.e., Belgium, Czech Republic, Denmark, Germany, Italy, Netherlands, Norway, Spain, Sweden, Slovenia, Switzerland and the UK) and non-Europe (i.e., Australia, Brazil, China, Egypt, Mexico, South Africa and the US).

Coming to 2) characteristics, we code:

e) *the type of data*. We distinguish between cross-sectional and longitudinal data. As noted by Werner and Woessman (2021), cross-sectional data do not allow to separate the Covid-19 effect from the cohort effects. Using this type of data, the performance of a cohort of students who have been affected by Covid-19 school closures is typically compared to the performance of a previous cohort of students who took the same test in a pre-Covid-19 period. However, this approach does not take into account the possibility that other factors influencing student achievement (e.g., change in education policies) might have changed coincidentally at the same time as Covid-19. Student-level longitudinal (panel) data help to address the cohort effects bias. They allow to look at changes in student performance before and after the lockdown and compare them with the progress made by similar students over the same period of previous years.

f) *the type of research design*. A number of different methodologies have been used in an attempt to identify the effect of Covid-19 school closures on academic achievement. In this study, we code the type of research design into the following three categories: descriptive, correlational, and quasi experimental/experimental (Locke et al., 2010). Studies using a descriptive research design (e.g., Moliner & Alegre, 2022) provide information about the average gap in test scores between the Covid-19 and non-Covid-19 cohorts without accounting for differences between these two cohorts (for example in terms of individual characteristics such as gender and socio-economic background) that could affect academic performances.^7^ On the other hand, studies employing a correlational research design (e.g., Ludewig et al., 2022) attempt to isolate the effect of Covid-19 from that associated with other factors that could influence student achievement, but their results cannot be given a causal interpretation. Finally, studies using a quasi-experimental or experimental design (e.g., Engzell et al., 2021) move closer to a causal interpretation of the relationship between Covid-19 and student performance.

g) *the publication year*. This study characteristic is a typical moderator variable in meta-analyses. It controls for time-trend effects (Schütt, 2021). In line with the approach followed by several recent meta-analyses (see, for instance, Di Pietro, 2022), we consider the year of the first appearance of a draft of the study in Google Scholar. This measure is preferred to publication year on the ground that journals significantly differ with respect to the time between online availability date of an article and the date when the article is given a volume and issue number^8^ (Al & Soydal, 2017). Additionally, in our dataset, there are two journal articles that are only available online and it is unclear in which issue of the journal they will be published. The publication years considered are: 2020, 2021, and 2022.

h) *the type of publication.* This moderator variable is considered in an attempt to control for the quality of the studies included in our sample. We distinguish between journal articles and other publication formats. Articles published in journals are expected to be of higher scientific rigour since they are more likely to have gone through a review process. Additionally, non-journal articles are more likely to contain typos in their regression tables (Cazachevici et al., 2020).

Finally, consistent with the approach taken in several studies (e.g., de Linde Leonard & Stanley, 2020), i) *the effect size's standard error* is also included among our moderator variables.

### 2.4 Sample characteristics

The dataset used for the meta-analysis includes a total of 239 different impact estimates extracted from 39 separate studies. Each study included in the dataset contains a number of estimates that vary from 1 to 32. Several reasons explain why most studies (i.e., 79%) reported multiple estimates. Many studies (e.g., Bielinski et al., 2021; Borgonovi & Ferrara, 2022; Feng et al., 2021; Gambi & De Witte, 2021; Maldonado & De Witte, 2022) estimated the effect of Covid-19 on student performance in several subjects. Similarly, a large number of studies (e.g., Ardington et al., 2021; Contini et al., 2021; Domingue et al., 2021; Gore et al., 2021) examined the impact of the pandemic on the achievement of students of different levels of education or even of students of different grades within the same level of education. For instance, Meeter (2021) analysed how Covid-19 affected the math performance of primary school children of grades 2–6. Some studies also provided different estimates showing both the short and long-term effects of Covid-19 on student achievement. For example, Kuhfeld et al. (2022) looked at changes in student test scores in fall 2020 and fall 2021 relative to fall 2019.

Table 1 presents the studies included in the dataset. Studies are listed alphabetically. For each study, we report information on the author(s), year of publication,^9^ country examined, type of test used to measure student performance, number of the effect sizes collected and their mean value.^10^ The studies cover a total of 19 countries. The largest source countries are the US (71 estimates), Germany (39 estimates) and Belgium (33 estimates).

**Table 1:** Sources for meta-analysis.

Study (Author(s) and year of publication)	Country	Type of test used to measure student performance	Number of effect sizes collected	Mean effect size
Ardington et al., 2021	South Africa	Individualstudent assessment administered by fieldworkers	4	−0.42
Arenas and Gortazar (2022)	Spain	Regional competency-based assessments	4	−0.04
Bielinski et al. (2021)	US	Adaptive assessment (FastBridge)	16	−0.14
Birkelund and Karlson (2021)	Denmark	Nationwide standardised tests	5	0.05
Borgonovi and Ferrara (2022)	Italy	Nationwide standardised tests	4	−0.04
Clark et al. (2021)	China	Standardised tests	1	0.22
Contini et al. (2021)	Italy	Standardised tests	2	−0.21
De Paola et al. (2022)	Italy	Local assessment at a single institution	1	−0.11
Depping et al. (2021)	Germany	Regional standardised tests	32	−0.01
Domingue et al. (2021)	US	Online reading assessment tool (Literably)	4	−0.03
El Said (2021)	Egypt	Local assessment at a single institution	1	−0.13
Engzell et al. (2021)	Netherlands	Standardised tests	4	−0.08
Fälth et al. (2021)	Sweden	Online assessment tool (LegiLexi)	18	0.09
Feng et al. (2021)	China	Large-scale exams administered by local governments	8	−0.50
Gambi and De Witte (2021)	Belgium	Standardised tests in the Flemish region	22	−0.13
Gore et al. (2021)	Australia	Progressive achievement tests administered by trained research assistants	4	0.04
Haelermans et al. (2021)	Netherlands	Standardised tests	3	−0.12
Hevia et al. (2022)	Mexico	Independent Assessment of Learning (MIA)	2	−0.54
Kofoed et al. (2021)	US	Local assessment at a single institution	5	−0.22
Kogan and Lavertu (2021a)	US	State assessment	1	−0.23
Kogan and Lavertu (2021b)	US	State assessment	11	−0.23
Korbel and Prokop (2021)	Czech Republic	Identical tests on a panel of 88 schools from all regions	2	−0.08
Kuhfeld et al.(2020)	US	Computer adaptive test (MAP Growth)	12	−0.10
Kuhfeld et al. (2022)	US	Computer adaptive test (MAP Growth)	24	−0.12
Lichand et al. (2021)	Brasil	Standardised tests in the São Paulo State	3	−0.31
Ludewig et al. (2022)	Germany	Progress in International Reading Literacy Study	2	−0.17
Maldonado and De Witte (2022)	Belgium	Standardised tests in the Flemish region	11	−0.16
Meeter (2021)	Netherlands	Digital learning assessment tool (Snappet)	10	0.15
Moliner and Alegre (2022)	Spain	Local assessment at a single institution	1	−2.34
Moliner et al. (2022)	Spain	Local assessment at a single high school	1	−0.95
Orlov et al. (2021)	US	Assessment of the same course across 4 institutions	2	−0.12
Rose et al. (2021)	UK	NFER assessments	6	−0.17
Schult et al. (2021)	Germany	Regional mandatory standardised tests	3	−0.06
Schuurman et al. (2022)	Netherlands	nationally standardised tests	2	−0.08
Skar et al.(2022)	Norway	Test administered by students at a single school	2	−0.48
Spitzer and Musslick (2021)	Germany	Assessment from an online mathematics platform (Bettermarks)	2	0.15
Tomasik et al. (2021)	Switzerland	Adaptive computer-based tool for formative student assessment (MINDSTEPS)	2	−0.07
van Der Velde (2021)	Netherlands	Assessment from an online retrieval practice tool used for language learning	1	0.25
Žnidaršič et al. (2022)	Slovenia	Local assessment at a single institution	1	0.11

Table 2 shows the descriptive statistics of the moderator variables used in the meta-regressions. While Column (1) displays simple averages (and standard deviations), Column (2) reports averages (and standard deviations) weighted by the inverse of the number of estimates reported in each study. Column (3) reports the number of effect sizes for each moderator variable.

**Table 2:** Descriptive statistics.

Variable name	Unweighted Mean (Standard deviation) (1)	Weighted (by the inverse of the number of estimates reported in each study) Mean (Standard deviation) (2)	Number of effect sizes (3)
Effect size (Cohen's *d*)	−0.112 (0.271)	−0.187 (0.436)	239
Effect size's standard error	0.021 (0.030)	0.035 (0.048)	239
*Subject*			
Math/Science	0.423 (0.495)	0.401 (0.491)	101
Humanities	0.527 (0.500)	0.456 (0.499)	126
Mix	0.050 (0.219)	0.143 (0.351)	12
*Level of Education*			
Primary	0.615 (0.488)	0.514 (0.501)	147
Secondary	0.343 (0.476)	0.359 (0.481)	82
Tertiary	0.042 (0.201)	0.127 (0.334)	10
*Timing of student assessment during Covid-19*			
2020	0.657 (0.476)	0.676 (0.469)	157
2021	0.343 (0.476)	0.324 (0.469)	82
*Geographical area*			
Europe	0.590 (0.493)	0.610 (0.489)	141
Non-Europe	0.410 (0.493)	0.390 (0.494)	98
*Year of publication*			
2020	0.126 (0.332)	0.127 (0.334)	30
2021	0.702 (0.458)	0.644 (0.480)	168
2022	0.172 (0.378)	0.229 (0.421)	41
*Type of data*			
Longitudinal	0.339 (0.474)	0.339 (0.474)	81
Cross-sectional	0.661 (0.474)	0.661 (0.474)	158
*Type of research design*			
Descriptive	0.130 (0.337)	0.242 (0.429)	31
Correlational	0.765 (0.424)	0.555 (0.498)	183
Quasi experimental/experimental	0.105 (0.307)	0.203 (0.403)	25
*Type of publication*			
Journal article	0.247 (0.432)	0.458 (0.499)	59
Other publication	0.753 (0.432)	0.542 (0.499)	180

### 2.5 Risk of bias assessment

In line with the approach adopted by Betthäuser et al. (2023) and Hammerstein et al. (2021), the risk of bias in nonrandomized studies was assessed in 38^11^ studies using the Risk Of Bias In Non-randomized Studies of Interventions (ROBINS-I) tool (Sterne et al., 2016). Each study was independently evaluated by the author and another researcher, and any disagreements were resolved through discussion to reach a consensus. Studies were scored on six different domains: confounding, participant selection, classification of interventions, missing data, measurement of outcomes, and reporting bias.^12^

Table 3 shows the risk of bias ratings for each domain (as well as an overall judgement) for the 38 studies. The lack of appropriate methods to control for confounders, sample selection problems and missing data appear to be the most common sources of potential bias. In several studies, vulnerable students, who have been among the most hardly hit by the pandemic, tend to be under-represented in the Covid-19 sample. This may lead to an underestimation of the pandemic-related learning delays. For example, the study by Gambi and De Witte (2021) relies on a sample where schools participating in the 2021 survey have a more advantaged student population in terms of neighbourhood of residence and mother's education, and have a smaller fraction of students that are considered to be slow learners. Similarly, in the longitudinal data used by Ardington et al., 2021 attrition is significantly higher for the Covid-19 group and attrition is associated with poorer pre-pandemic reading proficiency levels. In Kuhfeld et al. (2022), between fall 2019 and fall 2021, the number of students testing in a grade dropped significantly more in high-poverty schools compared to their low-poverty counterparts. In other studies, which use non-representative samples including convenience samples (e.g., Moliner & Alegre, 2022), the direction of the bias is unclear. One exception is the paper by Meeter (2021). In his sample the proportion of schools with a more disadvantaged student population appears to be slightly oversampled compared to all schools in the Netherlands, thus potentially biasing upwards the estimated impact of the pandemic on educational achievement. Finally, the question of how the use of non-appropriate methods to control for confounders might affect the estimated relationship between Covid-19 and student performance is addressed later when we discuss the results from the meta-regression analysis. As stated earlier, type of research design is one of our moderator variables.

**Table 3:** Risk of bias domain: ROBINS-I.

Study	Bias due to confounding	Bias in participant selection	Bias in classification of interventions	Bias because of missing data	Bias in measurement of outcomes	Bias in selection of the reported result	Overall risk of bias
Ardington et al., 2021	moderate	moderate	low	moderate	low	low	moderate
Arenas and Gortazar (2022)	Low	low	low	low	low	low	low
Bielinski et al. (2021)	moderate	moderate	low	low	low	low	moderate
Birkelund and Karlson (2021)	Low	low	low	low	low	low	low
Borgonovi and Ferrara (2022)	Low	low	low	moderate	low	low	moderate
Clark et al. (2021)	serious	serious	low	moderate	low	low	serious
Contini et al. (2021)	moderate	moderate	low	moderate	low	low	moderate
De Paola et al. (2022)	low	low	low	low	low	low	low
Depping et al. (2021)	serious	low	low	moderate	moderate	low	serious
Domingue et al. (2021)	moderate	moderate	low	moderate	low	low	moderate
El Said (2021)	serious	moderate	low	serious	low	low	serious
Engzell et al. (2021)	low	low	low	low	low	low	low
Fälth et al. (2021)	serious	moderate	low	serious	low	low	serious
Feng et al. (2021)	serious	serious	low	serious	low	low	serious
Gambi and De Witte (2021)	low	moderate	low	moderate	moderate	low	moderate
Gore et al. (2021)	moderate	low	low	low	low	moderate	moderate
Haelermans et al. (2021)	low	low	low	moderate	low	low	moderate
Hevia et al. (2022)	serious	serious	low	moderate	low	low	serious
Kogan and Lavertu (2021a)	moderate	moderate	low	moderate	low	low	moderate
Kogan and Lavertu (2021b)	low	moderate	low	moderate	low	low	moderate
Korbel and Prokop (2021)	serious	low	low	serious	low	low	serious
Kuhfeld et al.(2020)	moderate	low	low	moderate	low	low	moderate
Kuhfeld et al. (2022)	moderate	low	low	moderate	low	low	moderate
Lichand et al. (2021)	low	low	low	low	low	low	low
Ludewig et al. (2022)	moderate	low	low	low	low	low	moderate
Maldonado and De Witte (2022)	low	low	low	low	low	low	low
Meeter (2021)	serious	moderate	moderate	low	low	moderate	serious
Moliner and Alegre (2022)	serious	moderate	low	Serious	moderate	low	serious
Moliner et al. (2022)	serious	moderate	low	Serious	moderate	low	serious
Orlov et al. (2021)	moderate	low	low	moderate	low	low	moderate
Rose et al. (2021)	serious	serious	low	N/A	low	low	serious
Schult et al. (2021)	moderate	moderate	moderate	moderate	low	low	moderate
Schuurman et al. (2022)	serious	moderate	low	Serious	low	low	serious
Skar et al.(2022)	moderate	low	low	moderate	moderate	low	moderate
Spitzer and Musslick (2021)	serious	low	low	Low	low	moderate	serious
Tomasik et al. (2021)	serious	low	low	moderate	low	low	serious
Van der Velde et al. (2021)	serious	moderate	low	Low	moderate	low	serious
Žnidaršič et al. (2022)	moderate	moderate	low	moderate	serious	low	serious

### 2.6 Estimators and models

Two approaches frequently used in the meta-analysis literature are: 1) the Fixed Effects (FE) model, and 2) the Random Effects (RE) model. They rely on different assumptions. The FE model assumes that there is one true effect size common to all studies and that all differences in the observed effects can be attributed to within-study sampling error. By contrast, the RE model states that the effect size may vary between studies not only due to within-study sampling error, but also because there is heterogeneity in true effects between studies. Such additional variability is typically modelled employing a between-study variance parameter. Considering the characteristics of the studies included in our sample, it is difficult to assume that there is a common true effect that every study shares. Hence, it is anticipated that the RE model would be more suitable. Specifically, following the approach of Kaiser and Menkhoff (2020), we estimate the mean of the distribution of true effects using a RE meta-analysis based on a Robust Variance Estimation (RVE). The RVE approach allows to account for the possibility that multiple effect sizes from the same study are not independent from each other. The benefits of this method are that there is no need to drop any effect size (to ensure their statistical independency) and no information is required about the intercorrelation between effect sizes within studies.

In an attempt to investigate factors driving heterogeneity among effect sizes, a meta-regression model is estimated:

(1) $$T_{i}=\alpha +\sum_{n=1}^{N}\beta_{n}Z_{in}+\varepsilon_{i}$$

where $T_{i}$ denotes the estimated Cohen's *d* effect size, $Z_{in}$ is a vector of moderator variables, and $\varepsilon_{i}$ is the meta-regression disturbance term. The subscript i stands for the number of effect sizes included in the sample and the subscript n represents the number of moderator variables. In order to deal with the issue of heteroskedasticity in meta-regression analysis, we use Weighted Least Squares (WLS) with weights equal to the inverse of each estimate's standard error. This method is considered to be superior to widely employed RE estimators (Stanley & Doucouliagos, 2013).

A relevant problem in estimating equation (1) lies in the identification of the moderator variables to be included in the model. Selecting incorrect variables leads to misspecification bias and invalid inference (Xue et al., 2021). In line with several recent studies (e.g., Di Pietro, 2022; Gregor et al., 2021), the “general to specific” approach and the Bayesian Model Averaging (BMA) methodology are used to address model uncertainty. The advantages of the former method are that it addresses the issue of specification-searching bias and minimizes multicollinearity. Moderator variables are removed from the general specification in a stepwise fashion, dropping those with the largest *p*-value first until all the remaining variables are statistically significant. BMA is a method that runs many regressions containing different combinations of potential explanatory variables and weights them by model fit and complexity. Weighted averages of the estimated coefficients (posterior means) are computed using posterior model probabilities (akin to information criteria in frequentist econometrics). Each coefficient is also given a Posterior Inclusion Probability (PIP), which is the sum of posterior model probabilities of the models including the relevant variable and indicates how likely such a variable is to be contained in the true model (Havránek et al., 2018).

### 2.7 Publication bias

Publication bias has long been identified as a major problem in meta-analysis (Dwan et al., 2008). Such an issue occurs because editors and scholars tend to prefer publishing papers with statistically significant or non-controversial results. This may lead to distorted conclusions as published findings may end up overstating the true effect. Evidence of publication bias has been found in meta-analyses covering different fields (see, for instance, Begg and Belin (1988) in the case of medical studies).

**Fig. 2:**
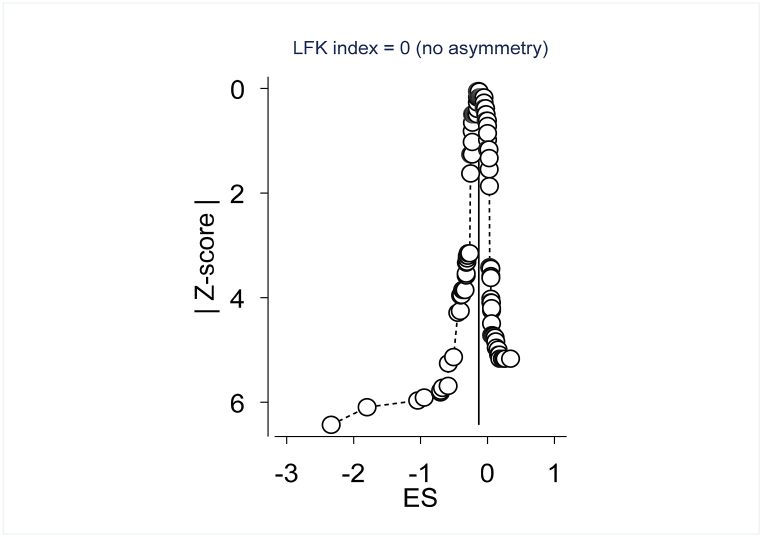
Doi plot.

In line with previous studies (e.g., Di Pietro, 2022), we use the Doi plot to graphically evaluate publication bias. Not only does the Doi plot enhance visualization of the asymmetry (in absence of publication bias there is no asymmetry), but it also allows for measuring the asymmetry through the Luis-Furuya-Kanamori (LFK) index. LFK index values within ±1 suggest no asymmetry, LFK index values exceeding ±1 but within ±2 indicate minor asymmetry, while LFK index values exceeding ±2 denote major asymmetry (Furuya-Kanamori et al., 2018). As shown in Fig. 2, the Doi plot shows no asymmetry (LFK index = 0), indicating that no publication bias is detected.

To further examine the risk of publication bias, we employ the Egger's test (Egger et al., 1997) where the effect size is regressed against its precision (indexed by its standard error). Results indicate that we can safely accept the null hypothesis of no publication bias (*p*-value = 0.380).

Our findings are consistent with those in previous relevant meta-analyses. König and Frey (2022) as well as Betthäuser et al. (2023) conclude that the presence of publication bias is unlikely.

## 3 Results and discussion

This Section is divided into three parts: first, we estimate a summary effect size (Section 3.1.); second, we investigate potential sources of heterogeneity (Section 3.2.); and third we provide a discussion of the main results (Section 3.3.).

### 3.1 Summary effect size

In order to calculate the overall summary effect, we fit an intercept-only RE RVE model to our set of effect sizes. In such a model, the intercept can be interpreted as the precision-weighted mean effect size adjusted for effect-size dependence (Friese et al., 2017).

The RVE RE mean effect size turns out to be −0.186^13^ (SE = 0.0646, *p*-value = 0.0065, 95% CI [-0.316, −0.055]). It is also important to note that in this model the small-sample corrected degrees of freedom is greater than 4 (i.e., 39), suggesting that the *p*-value for the associated *t*-test accurately reflects the type I error (Tanner-Smith et al., 2016).

Next, we compute the I^2^ statistic to assess the heterogeneity of the results across studies (Higgins et al., 2003). The appropriateness of the RE model is confirmed as I^2^ has a value of 100%.^14^ This suggests that all the variability in the effect-size estimates is due to heterogeneity as opposed to sampling error. Additionally, we also look at $\tau^{2}$ (between-study variance),^15^ which denotes the variability in the underlying true effects. Its large value of 1.74 further corroborates the hypothesis of substantial heterogeneity of the effect sizes (Takase & Yoshida, 2021).

One should observe that our findings from the RVE analysis are broadly consistent with those from previous meta-analyses. Storey and Zhang (2021a) concluded that due to Covid-19 students lost, on average, 0.15 standard deviations of learning, König and Frey (2022) found average losses of 0.175 standard deviations, and Betthäuser et al. (2023) estimated average losses at 0.14 standard deviations.^16^ Two considerations help put these results into perspective. First, one may notice that the delayed learning suffered by students as a result of Covid-19 school closure is roughly comparable to that experienced by their peers after major natural disasters. For instance, Sacerdote (2012) found that in the spring of 2006 students who were displaced by Katrina and Rita hurricanes saw their test scores fall by between 0.07 and 0.2 standard deviations. A similar result, though of a smaller magnitude, is obtained by Thamtanajit (2020). He showed that in Thailand floods reduced student test scores by between 0.03 and 0.11 standard deviations, depending on the subject and educational level. Second, following Hanushek and Woessmann (2020), a learning deficit of about 0.186 standard deviations can be considered to be equivalent to the loss of just over half of a school year.^17^

While our results suggest that the pandemic lowered student performance on average by about 0.19 standard deviations, there is a large consensus that it did not affect students equally. For instance, several studies (see, for example, Engzell et al., 2021; Hevia et al., 2022) showed that Covid-19 had a detrimental effect especially on the achievement of students from less advantaged backgrounds. During school closures, these students are less likely to have had access to a computer, an internet connection, and a space conducive to learning (Blaskó et al., 2022; Di Pietro et al., 2020). Moreover, as argued by Ariyo et al. (2022), one would expect children of less educated parents to have received less parental support while learning at home than children of more educated parents. Greenlee and Reid (2020) provide evidence on this, showing that in Canada during the pandemic the frequency of children's participation in academic activities increased with parental educational levels.

### 3.2 Heterogeneity

Table 4 shows the results of regressing our standardised measure of student achievement against the moderator variables described above. Column (1) of Table 4 presents estimates from a regression where all potential explanatory variables are included. However, including all 13 variables (in addition to the constant term) in the regression may inflate standard errors and lead to inefficient estimates given that some of the variables may turn out to be redundant. Therefore, the “general-to-specific” approach is employed in an attempt to identify the influential factors. Following this strategy, as shown in Column (2) of Tables 4, 6 independent variables (in addition to the constant term) are included in the model. To account for the potential dependence of multiple estimates reported by a given study, in Column (3) of Table 4 standard errors are clustered at the study level. Furthermore, since there are relatively few clusters (i.e., 39), following Cameron and Miller (2015) we apply the correction for small number of clusters by employing wild score bootstrapping (Kline & Santos, 2012). Estimates shown in Column (3) indicate that a few moderator variables are robustly important. In line with expectations, students experienced larger learning deficits in math/science. More precisely, other things being equal, student achievement in math/science is on average found to be 0.17 standard deviations smaller than in humanities/subject mix. Our findings indicate also that the negative effect of Covid-19 on student achievement appears to be more pronounced when using experimental/quasi experimental techniques than when using descriptive or correlational research designs. Additionally, studies employing cross-sectional data as well as those focusing on non-European countries tend to suggest greater learning deficits.

**Table 4:** Meta-regression results.

	General model (1)	Specific model (2)	Robust Specific model (3)	Robust Specific model (using the inverse of the variance as weight) (4)
Constant	−0.119 (0.175)	−0.173*** (0.049)	−0.173*** (0.032) [0.000]	−0.207*** (0.055) [0.051]
*Subject (Reference category: Humanities)*				
Math/Science	−0.170*** (0.008)	−0.170*** (0.007)	−0.170*** (0.008) [0.000]	−0.180*** (0.000) [0.000]
Mix	−0.113 (0.144)			
*Level of education (Reference category: Primary)*				
Secondary	0.097*** (0.008)	0.097*** (0.008)	0.097*** (0.007) [0.298]	0.102*** (0.000) [0.334]
Tertiary	0.142 (0.292)			
*Timing of student assessment during Covid-19 (Reference category: 2020)*				
2021	0.080 (0.066)			
*Geographical area (Reference category: non-Europe)*				
Europe	0.180*** (0.068)	0.193*** (0.051)	0.193*** (0.034) [0.002]	0.244*** (0.055) [0.013]
*Year of publication (Reference category is: 2022)*				
2020	0.013 (0.058)			
2021	−0.032 (0.095)			
*Type of data* (*Reference category: cross-sectional)*				
Longitudinal	0.079 (0.109)	0.141*** (0.049)	0.141*** (0.032) [0.020]	0.178*** (0.055) [0.153]
*Type of research design* (Reference category: descriptive)				
Correlational	−0.085 (0.131)			
Quasi experimental/experimental	−0.223 (0.170)	−0.228*** (0.050)	−0.228*** (0.029) [0.002]	−0.205*** (0.055) [0.005]
*Type of publication (Reference category: Other publication)*				
Journal article	−0.110** (0.044)	−0.097** (0.041)	−0.097*** (0.018) [0.235]	−0.143*** (0.005) [0.646]
Standard Error	−0.194 (2.834)			
R-squared	0.747	0.742	0.742	0.792
No. observations	239	239	239	239

*Note.* Standard errors are in parentheses. Standard errors are clustered at study level (39 clusters) in Columns (3) and (4). In square brackets we report score wild cluster bootstrap *p*-values (Kline & Santos, 2012) generated using boottest command in Stata with 999 replications (Roodman, 2016). In Columns (1), (2), and (3) the regressions are estimated by weighted least squares where each effect size estimate is weighted by its inverse standard error. In Column (4), the regression is estimated by weighted least squares where each effect size estimate is weighted by its inverse variance.

*, **, and *** denote statistical significance at 10, 5, and 1%, respectively.

As a robustness test, the model depicted in Column (3) of Table 4 is re-estimated but this time each effect size is weighted by its inverse variance. As shown in Column (4) of Table 4, with the exception of the estimate on longitudinal data, the sign and the magnitude of the other coefficients are broadly in line with those depicted in Column (3).

Next, the BMA approach is employed as an alternative to address the problem of uncertainty in the specification of the meta-regression model.^18^ In BMA, following the rule of thumb proposed by Kass and Raftery (1995), the significance of each explanatory factor is considered not to be weak if the PIP is larger than 0.5. The results, which are reported in Table 5, show that all the variables that are consistently identified by the BMA methodology as relevant (i.e., *Math/Science*, *Europe* and *Journal article*) are also included in the specification whose estimates are reported in Columns (2), (3) and (4) of Table 4. Although the PIP associated with *Quasi experimental*/*experimental* does not quite make the relevant threshold, it is relatively close to it.

**Table 5:** Bayesian model averaging (BMA).

	BMA		
	Post mean	Post St. error	PIP
Constant	−0.059	0.106	1.00
**Math/Science**	−0.150	0.009	1.00
Mix	−0.137	0.152	0.50
Secondary	0.535	0.980	0.29
Tertiary	0.009	0.098	0.07
2021 (Timing of student assessment during Covid-19)	0.011	0.037	0.13
**Europe**	0.074	0.092	0.73
2020 (Year of publication)	0.010	0.036	0.17
2021 (Year of publication)	−0.007	0.042	0.16
Longitudinal	0.003	0.055	0.22
Correlational	0.021	0.073	0.15
Quasi experimental/experimental	−0.110	0.139	0.44
**Journal article**	−0.102	0.091	0.64
Standard Error	−0.124	1.070	0.07

### 3.3 Discussion of the main results

Our meta-analysis delivers six main results.

First, we find that, on average, the pandemic depressed student achievement by around 0.19 standard deviations. While this result is in line with the conclusions of earlier meta-analyses and systematic reviews, it should be taken into account that we use a more balanced sample in terms of country composition. This would suggest that our finding is more generalizable than that of previous studies.

Second, the pandemic caused a larger learning deficit in math/science compared to other subjects. This means that extra-support in math/science may be especially needed to help students catch up following the disruption caused by Covid-19.

Third, the effect of Covid-19 on student achievement does not appear to statistically differ across levels of education. Consistent with the findings of Betthäuser et al. (2023), our results suggest that pandemic-related learning delays are similar across primary and secondary school students. In addition, this research has shown that these learning delays are not statistically different from the learning deficits suffered by tertiary education students. While, as discussed in Subsection 2.3.2, one would have expected Covid-19 school closures to have had a more negative impact on the achievement of younger students than older students, this effect could have been offset by the greater support in terms of parental involvement received by the former group of students during online learning. Bubb and Jones (2020) found that in Norway, during the peak of the Covid-19 lockdown period, the proportion of parents/carers who reported having gained more information about their children's learning was higher in lower grades than in higher grades. Besides learners' age considerations, one should also observe that the shift towards online learning could have had a detrimental impact on the knowledge and skills of those students, mainly at secondary and tertiary levels, whose curriculum includes experiential learning experiences (e.g., field trips, hands-on activities) that cannot take place virtually (Tang, 2022). However, at the same time, given that our analysis was not conducted at grade level, one cannot rule out the possibility that the pandemic has disproportionately affected the achievement of very young pupils (e.g., grade 1). In other words, there could be heterogeneity within primary school children.

Fourth, our results indicate that in 2021 students were not able to recover from the learning deficits caused by Covid-19 school closures in 2020. There is no statistically significant difference in student performance between assessments that have taken place several months or more than one year after the outbreak of the coronavirus and those that have occurred in the early stages of the pandemic. A similar finding has been obtained by Betthäuser et al. (2023). It is important to note that, if not addressed, the learning deficits suffered by students may result in significant long-term consequences. Without remedial education upon school re-opening, not only may students who have been disproportionately affected by the pandemic continue to fall behind, but their learning achievements may also suffer a further setback as time goes on (Angrist et al., 2021). Kaffenberger (2021) estimates that if learning in grade 3 is reduced by one-third, the equivalent of about a three-month school closure, learning levels in grade 10 would be a full year lower. Özdemir et al. (2022) forecast that the pandemic could erase decades-long gains in adult skills for affected cohorts unless interventions to alleviate learning deficits are quickly implemented. Additionally, several papers show that there is a relationship between test scores and labour market performance. For instance, Chetty et al. (2014) find that raising student achievement by 0.2 standard deviations is expected, on average, to increase annual lifetime earnings by 2.6%.

Fifth, the extent of the learning deficit seems to be smaller among students in Europe relative to their peers in the rest of the world. Although the reasons behind such a result are unclear, this might be due to several factors. First, one should note that the European countries considered in this study have, on average, a higher gross domestic product per capita than most of the non-European countries included in the analysis (this is not true for the US and Australia). As suggested by Donnelly and Patrinos (2020), high-income countries are likely to have experienced smaller learning deficits as a result of Covid-19 because of their higher technological capability and the lower share of households living below the poverty line.^19^ Second, Schleicher (2020) observes that the impact of the virus on education might have been less severe in many European countries and Southern Hemisphere countries whose 2019–2020 academic calendars had scheduled breaks (up to two weeks) that fell within the school closure period due to Covid-19. Third, there is evidence, but only available at higher education level, that European educational institutions were better prepared to respond to the challenges posed by the pandemic than their counterparts in other parts of the world. A survey carried out by the International Association of Universities immediately after the outbreak of the coronavirus shows that the percentage of higher education institutions where classroom teaching was replaced by distance teaching and learning was higher in Europe than in other continents (Marinoni et al., 2020).

Sixth, our findings seem to suggest that studies using non-causal methods tend to underestimate the negative effect exerted by Covid-19 on student performance. The study by Betthäuser et al. (2023) also hints at the same conclusion, but their meta-analysis does not provide any evidence on this. As pointed out by Engzell et al. (2021), non-causal methods fail to account for trends in student progress prior to the outbreak of Covid-19 and, hence, by assuming a counterfactual where achievement has stayed flat, they generate estimates of learning deficits that are biased downwards. The underestimation of pandemic-related learning delays may have important policy implications as it could result in under-provision of remedial support to students who are falling behind due to Covid-19.

## 4 Conclusions

We have assembled and studied a new sample of estimates about the impact of Covid-19 on student achievement. The sample includes 239 estimates from 39 studies covering 19 countries. One of the key findings emerging from our study is that the detrimental effects of Covid-19 school closure on student learning appear to be long-lasting. This calls for more efforts to help students recover from missed learning during the pandemic. As initiatives and programs aimed at learning recovery can be quite costly, several researchers (e.g., Patrinos, 2022) stress the importance of protecting the education budget whilst considering the competing financial needs of other sectors such as, for instance, health and social welfare (UNESCO, 2020b). Therefore, given the current policy climate where public resources are in high demand by various sectors, it is more important than ever to identify and adopt cost-effective measures.

While there seems to be a relatively large consensus in the literature that small group tutoring programs are a cost-effective way to mitigate the learning deficits caused by the pandemic (see, for instance, Burgess, 2020; Gortazar et al., 2022), less attention has been paid to a number of time- and cost-effective pedagogical practices (Carrasco et al., 2021). Promoting the development of metacognition skills is, for instance, a powerful way to enhance student learning and performance (Stanton et al., 2021). Metacognition allows students to think about their own learning, and this may increase their self-confidence and motivation. Similarly, increased collaboration and dialogue between students can support learning. Peers may help students clarify study materials and develop critical thinking. Overall, a better understanding is needed about the different types of educational interventions available and their cost-effectiveness. It would be desirable if governments at national, regional and local levels could exchange their experiences in this field and learn from each other.

## Funding details

This work has not been supported by any grants.

## CRediT authorship contribution statement

**Giorgio Di Pietro:** Conceptualization, Data curation, Formal analysis, Investigation, Methodology, Software, Writing – review & editing.

## Declaration of competing interest

No potential conflict of interest was reported by the author.

## Data Availability

Data will be made available on request.

## Footnotes

2 In this study, the term “learning deficit” refers to the lower learning outcomes achieved by students due to the pandemic relative to what would have been expected if the pandemic had not occurred.

3 Previous meta-analyses include König and Frey (2022) who extracted 109 effect sizes nested in 18 studies, Storey and Zhang (2021a) who synthetized 79 effect sizes from 10 studies, and Betthäuser et al. (2023) who considered 291 effect sizes from 42 studies. The reviews by Patrinos et al. (2022), Moscoviz and Evans (2022), Donnelly and Patrinos (2022), Hammerstein et al. (2021) and Zierer (2021) summarised the results of 35 studies, 29 studies, 8 studies, 11 studies and 9 studies, respectively.

4 Similarly, in Storey and Zhang (2021a) 7 out of the 10 studies considered in the meta-analysis are from the US or the UK.

5 One should, however, bear in mind that studies from high-income countries are strongly over-represented.

6 For Google Scholar, in line with the approach of Romanelli et al. (2021), only the first 100 relevant references at each search were retrieved, as results beyond the first 100 entries were largely irrelevant given the purpose of this study.

7 These studies typically report in a table the mean test scores of the Covid-19 and non-Covid-19 cohorts, together with their corresponding standard deviations and information about the respective sample sizes of the two cohorts. Mean test scores ($X_{1}$, $X_{2}$) and their standard deviations ($S_{1}$, $S_{2}$) can be used to compute the Cohen's *d* (i.e., $\frac{\left(X_{2}-X_{1}\right)}{\sqrt{\frac{\left(S_{1}^{2}+S_{2}^{2}\right)}{2}}}$). Next, Cohen's *d* standard error can be computed using the formula given in Cooper and Hedges (1994) where information about the sample sizes of the two cohorts and the estimated Cohen's *d* are used.

8 For instance, in our sample, the journal article by Maldonado and De Witte was available online in 2021 but was published in 2022. On the other hand, the journal article by Ardington et al. was available online and published in 2021.

9 In this table, we report the actual year of publication of the latest version of the study (for journal articles this is the year when they are assigned a volume and issue number) rather than the year of the first appearance of a draft of the study in Google Scholar.

10 All the extracted effect sizes and their standard errors can be found in the supplementary Appendix.

11 One of the studies included in our sample (i.e., Kofoed et al., 2021) does use a randomized design.

12 Following Betthäuser et al. (2023), the domain “deviation from intended interventions” was not considered. As noted by Hammerstein et al. (2021), information on this domain is very rarely included in the relevant studies because Covid-19 school closures were not intended interventions.

13 The *robumeta* command in Stata is employed. An intercept-only model is run where the estimate of the meta regression constant is equal to the unconditional mean effect size across studies. With this command, it is possible to specify a value for *rho*, the expected correlation among dependent effects. Following Tanner-Smith and Tipton (2013), we use different values of *rho* ranging from 0 to 1 in intervals of 0.1 in an attempt to check the consistency of results. All models yield the same outcome regardless of the specified value of *rho*.

14 A value of I^2^ greater than 75% is considered large heterogeneity (Higgins et al., 2003).

15 This is calculated using the method-of-moments estimator provided in Hedges et al. (2010).

16 Relevant systematic reviews have also found similar learning deficits. Donnelly and Patrinos (2022) found average delays of 0.13 standard deviations, Zierer (2021) estimated average losses at 0.14 standard deviations, and Hammerstein et al. (2021) reported average deficits of 0.10 standard deviations.

17 They found that the loss of one third of a school year of learning is equivalent to approximately 11% of a standard deviation of lost test results. This finding is broadly consistent with that obtained by Hill et al. (2008) who conclude that a value of Cohen's *d* of 0.4 (with a margin of error of ±0.06) corresponds to the average annual reading achievement gains in fourth grade.

18 We treat all moderator variables as auxiliary covariates while the constant is treated as a focus regressor. Each effect size is weighted by its inverse standard error.

19 Results from the meta-analysis by Betthäuser et al. (2023) support this proposition.
